# Noonan syndrome spectrum disorders in real life: patient characteristics and response to growth hormone therapy in a genetically defined single-country multicenter cohort

**DOI:** 10.1007/s00431-026-06764-2

**Published:** 2026-01-24

**Authors:** Barbora Jirova, Maria Najdekova, Jana Cerna, Petra Dusatkova, Kristina Holotova, Stanislava Kolouskova, Ivana Kotvalova, Olga Magnova, Martin Modrak, Dana Novotna, Barbora Obermannova, Jan Pavlicek, Lukas Plachy, Renata Pomahacova, Stepanka Pruhova, Jitka Rezabkova, Jiri Strnadel, Marta Snajderova, Zdenek Sumnik, Jirina Zapletalova, Jan Lebl

**Affiliations:** 1 Department of Paediatrics, 2nd Faculty of Medicine, Charles University, Prague, Czechia; 2 Department of Paediatrics, Motol and Homolka University Hospital, Prague, Czechia; 3 Department of Paediatrics, Faculty of Medicine, University of Ostrava and University Hospital, Ostrava, Czechia; 4 Department of Paediatrics, Faculty of Medicine, Palacky University and University Hospital, Olomouc, Czechia; 5 Department of Paediatrics, Hradec Kralove, Faculty of Medicine, Charles University and University Hospital, Hradec Kralove, Czechia; 6 Department of Paediatrics, Faculty of Medicine, Masaryk University and University Hospital, Brno, Czechia; 7 Department of Bioinformatics, 2nd Faculty of Medicine, Charles University, Prague, Czechia; 8 Department of Paediatrics, Plzen, Faculty of Medicine, Charles University and University Hospital, Plzen, Czechia

**Keywords:** Noonan syndrome spectrum disorders, Growth hormone therapy, Short stature, Genetic testing, *PTPN11*, *SOS1*

## Abstract

The variety of genes associated with Noonan syndrome spectrum disorders (NSSD) is expanding, and real-life experience with its management is increasing; however, phenotypic differences among genotypes remain poorly defined. We aimed to assess clinical characteristics and response to growth hormone (GH) therapy in a genetically confirmed, single-country multicentre NSSD cohort. We included 101 patients with NSSD (56 males) from six centres: 76 with (likely) pathogenic *PTPN11* variants, 7 with *SOS1* variants, and 18 with other gene variations. All completed at least one year of GH therapy; 23 reached final height. Parental heights were below average (fathers: − 0.33 SDS [− 1.19; 0.39], *p* < 0.01; mothers: − 0.68 SDS [− 1.47; 0.12], *p* < 0.001; medians [IQR]). SOS1-NS patients were born earlier (gestational week 36 [31; 37]) compared to *PTPN11*-NS. Birth length (− 1.23 SDS [− 1.74; − 0.57]) was more reduced than weight (− 0.29 SDS [− 1.10; 0.54]; *p* < 0.0001); *PTPN11-*NS*/SOS1*-NS had the lowest birth weights (*p* < 0.05). GH was started at 6.4 years (3.8; 9.5), with baseline height-SDS − 2.92 (− 3.64; − 2.47). Median annual height-SDS increments were similar across genotypes: 0.61 (year 1; *n* = 101), 0.28 (year 2; *n* = 92), 0.21 (year 3; *n* = 77), 0.07 (year 4; *n* = 63), and 0.09 (year 5; *n* = 41), leading to height-SDS − 1.97 (− 2.81; − 1.42) after 5 years. Menarche occurred at age 15.7 (13.8; 17.2) years (*n* = 13), and final height-SDS (available in 23 patients) reached − 1.68 (− 2.65; − 0.41).

*Conclusions* Growth restriction in NSSD begins prenatally, especially in PTPN11-NS and SOS1-NS. GH therapy was associated with improved height SDS, with the largest height gains observed before puberty. Earlier treatment initiation may therefore be beneficial for growth outcomes.

**Table:** 

**What is Known:** • *Noonan syndrome spectrum disorders (NSSD) are genetically heterogeneous, with pathogenic variants in the PTPN11 and SOS1 genes being most prevalent. Phenotypic differences among genotypes remain poorly defined.* • *Short stature is a key NSSD feature. Growth hormone (GH) therapy is beneficial, but prior studies lacked genetic justification or had limited sample sizes.*	
**What is New:** • *We analysed perinatal data and real-life GH outcomes in 101 genetically confirmed NSSD cases.* • *SOS1-NS was linked to prematurity. Birth length was more reduced than weight across genotypes; PTPN11/SOS1 cases had the lowest birth weights. GH therapy was associated with an increase in height SDS from − 2.92 to − 1.97 (median) following 5 years, and to − 1.68 in those with final height.*

**Supplementary Information:**

The online version contains supplementary material available at 10.1007/s00431-026-06764-2.

## Introduction

Noonan syndrome (NS; [MIM 163950]) was first recognised by paediatric cardiologist Jacqueline Noonan, who noted a recurring constellation of congenital heart defects, short stature, hypertelorism, and characteristic facial features, later formalised in 1968 [1, 2]. With an incidence of ~ 1:1000–1:2500 live births, NS ranks among the most prevalent dysmorphic syndromes [3]. Since the discovery of pathogenic variants in the protein tyrosine phosphatase non-receptor-type 11 (*PTPN11*) gene, which encodes the protein tyrosine phosphatase SHP2 in 2001 [4], extensive genetic heterogeneity has been described in NS, involving multiple genes within the RAS/MAPK pathway. Early molecular studies documented *PTPN11* causal gene variants among clinically defined cohorts [5–9], and subsequent work in similarly defined populations reported comparable distributions [10–13]. Findings from fully genotyped cohorts have corroborated these patterns across settings [14–16]. Over the following years, numerous additional causative genes involved in the RAS/MAPK signalling cascade have been identified, including *SOS1*, *SOS2*, *KRAS*, *RAF1*, *NRAS*, *RIT1*, *SHOC2*, *BRAF*, *LZTR1*, and others [17], with recent single-country cohorts further expanding the spectrum by reporting rarer loci and novel variants [18]. Variants in these genes result in a group of related clinical conditions collectively known as RASopathies or Noonan Syndrome Spectrum Disorders (NSSD), which share overlapping clinical features due to dysregulation of the same intracellular signalling pathway. The RASopathies family includes NS, NS with multiple lentigines (formerly referred to as LEOPARD syndrome), Noonan-like syndrome with loose anagen hair, cardiofaciocutaneous (CFC) syndrome, and Costello syndrome [19].

Growth impairment is one of the hallmark clinical features across NSSD, frequently resulting from disruptions that interfere with normal growth at multiple levels. Short stature in NS typically results from impairment across multiple stages of growth: variably reported prenatal growth restriction, with several cohorts documenting slightly reduced birth length [14–16, 20, 21], followed by reduced early childhood growth spurt, delayed puberty with diminished acceleration, and ultimately a below-average adult height attained later than in the background population. Beyond endocrine factors, early feeding difficulties may also contribute to growth failure in NSSD, with first-year deceleration only partly explained by feeding problems [22], and targeted behavioural therapy for avoidant/restrictive food intake disorder improving intake and weight/height indices in NSSD [23]. Recombinant human growth hormone (GH) therapy has been widely used to improve height outcomes in NSSD [24]. Several studies suggested that GH responsiveness may vary by genotype. Binder first highlighted that children with *PTPN11* variants, present in about half of all NS cases, tend to show smaller short-term gains in height SDS than variant-negative peers [25]. This observation is biologically plausible, as the SHP2 protein encoded by *PTPN11* attenuates GH receptor signalling by dephosphorylating STAT5, resulting in a form of mild GH insensitivity. Subsequent genotyped cohorts provided mixed results: in some, prepubertal children with *PTPN11* variants showed lower increases in growth velocity or Δheight-SDS, while *SOS1* carriers often started taller and tracked more favourably during childhood [16, 21, 26]. In contrast, other reports found no significant differences in long-term or adult height outcomes between genotypes, although these analyses were typically underpowered [15, 27]. Taken together, existing evidence suggests possible genotype-related trends, but results remain inconsistent due to small subgroup sizes and heterogeneous study designs.

Although molecular diagnostics have become increasingly accessible, much of the available literature on growth patterns and GH outcomes in NS is derived from clinically diagnosed cohorts without genetic confirmation [28–31]. This heterogeneity limits the interpretation of genotype-specific growth characteristics, whereas genetically stratified analyses allow more accurate correlations between molecular findings and clinical outcomes [25]. To date, however, no large real-world, registry-based cohort has provided robust genotype-stratified GH outcome data in NSSD. Such information is clinically needed to provide realistic counselling for families, optimise therapeutic timing and monitoring, and establish reliable benchmarks. It may also serve as a reference when evaluating novel targeted approaches to RAS/MAPK dysregulation, such as SHP2 or MEK inhibitors and C-type natriuretic peptide analogues [32]. To address this gap, we analysed a genetically defined, multicentre cohort of Czech children with NSSD. Leveraging longitudinal growth data stratified by specific variants, we examined differences from birth through postnatal development and during GH therapy. Our objective was not to prescribe gene-specific treatment algorithms, but to clarify genotype-related variability and provide evidence to informed clinical counselling.

## Patients and methods

### Study design and participants

This was a retrospective, multicentre cohort study based on data primarily derived from the national registry of paediatric growth hormone recipients (REPAR) and subsequently verified by the respective attending paediatric endocrinologists.

As of December 31 st, 2024, the REPAR [33] database included records of 6009 paediatric patients treated with GH in the Czech Republic. These data comprised both current patients (entered prospectively since 2014 by attending paediatric endocrinologists) and historical records transferred from previous international registries (KIGS, Nordinet, GeNeSIS, and ECOS; covering the period from 1992 to 2014).

**Fig. 1:**
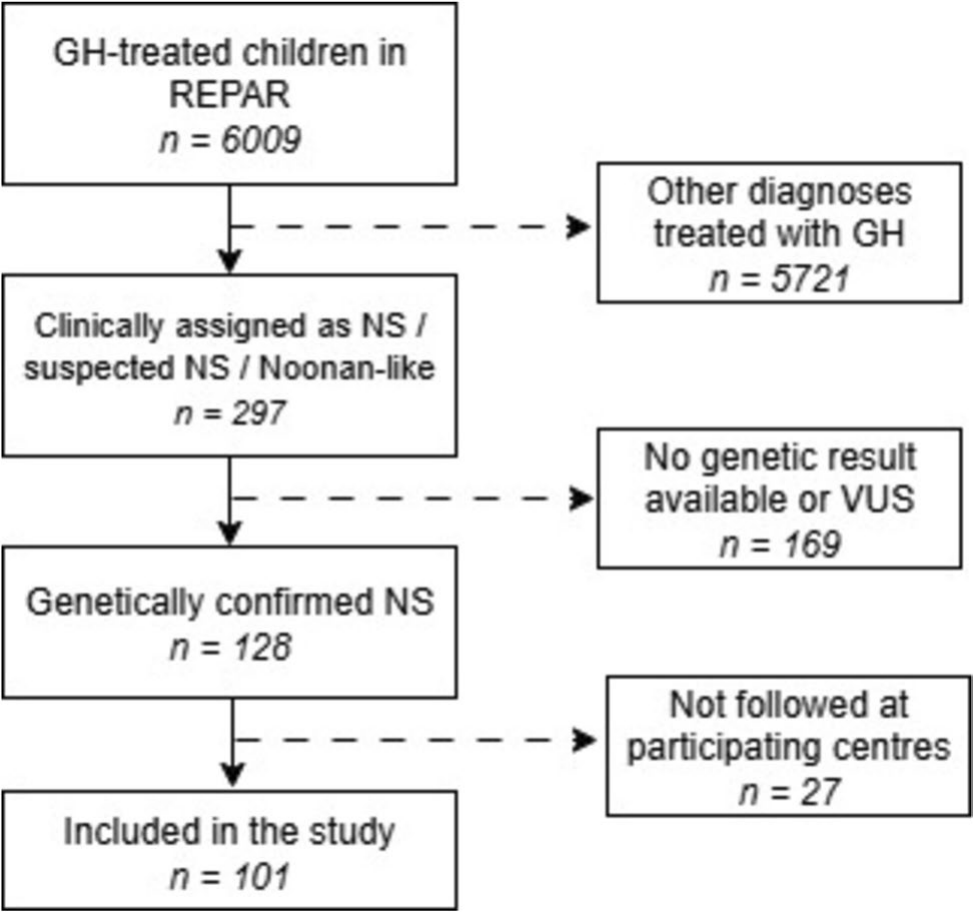
Patient selection from the REPAR registry. Dashed arrows indicate excluded cases. GH: growth hormone, NS: Noonan syndrome, VUS: variants of unknown significance

Within this registry, 297 children had been classified as having NS, suspected NS, or a NS-like phenotype, based either on the clinical diagnostic criteria proposed by van der Burgt [34] or on an already known genetic diagnosis prior to referral. In 169 cases, either no genetic testing was performed, or the result was unavailable, or the identified variant was not classified as pathogenic (P) or likely pathogenic (LP). The remaining 128 patients had a genetic result confirming the diagnosis of NSSD (P or LP variant in a gene known to be associated with Noonan syndrome). Of these, 101 (56 males) already completed at least one year of GH treatment and were followed at one of six major paediatric endocrinology centres that agreed to participate in the study (Fig. 1). These centres jointly overlook 82% of all children currently receiving GH therapy nationwide [33]. GH therapy is available for all children with residency in the country who comply with the indication criteria, including diagnosis of NS and other NSSD, and is fully reimbursed by public health insurance—thus, selection bias due to social-economic status is improbable.

Among the final study cohort, the distribution of confirmed P or LP variants was as follows: *PTPN11* (*n* = 76), *SOS1* (7), *RAF1* (4), *KRAS* (3), *BRAF* (2), *HRAS* (2), *SOS2* (2), *SHOC2* (2), *LZTR1* (1), *MAP2K1* (1), and *NRAS* (1). The list of detailed gene variants is available in Supplementary Table (Tab. S1).

### Clinical data

Clinical data as collected in the REPAR database, and subsequently verified for this study, include parental heights, gestational week of delivery, birth weight and length, height at initiation of GH therapy, annual height measurements at years 1 to 5 of GH administration according to the individual duration of GH therapy at the timepoint of data collection for this study, and pubertal status. In participants who underwent GH stimulation testing, peak GH levels were recorded. In those who already reached pubertal milestones, we evaluated age and height at first signs of puberty, age, and height at menarche in girls, and final height. Birth length and weight data were converted to standard deviation scores (SDS) for appropriate gestational week [35] and heights during childhood, puberty, and final heights were converted to SDS based on Czech national growth references [36]. Heights of parents were expressed as SDS of national standards by the age of 18 years. Final height was defined as the height reached when annual growth velocity declined to less than 1 cm/year, indicating cessation of significant longitudinal growth, in individuals with full pubertal maturation. All study subjects were treated with GH administered as daily subcutaneous injections. The median prescribed daily GH dose as recorded in the REPAR database was 0.038 mg/kg body weight (0.030–0.055; P5–95) and fluctuated mildly within the course of therapy [33]. Only children with NSSD and a height-SDS below − 2 were considered eligible for GH therapy, in accordance with the currently approved indication criteria. The decision to initiate GH therapy in each individual child was at the discretion of the attending paediatric endocrinologist, following a discussion of the pros and cons with the parents or other legal guardians.

### Genetic testing

Genetic analyses were conducted across a span of approximately two decades, reflecting the evolution of known genes causative of NSSD, and of molecular genetic diagnostic techniques. Initially, Sanger sequencing of *PTPN11* became the diagnostic standard, stepwise accompanied by additional newly identified genes. As next-generation sequencing (NGS) technologies matured in the early 2010 s, multigene panels targeting RASopathy-associated genes were increasingly implemented, offering broader and more efficient genetic screening. In the mid to late 2010 s, Whole Exome Sequencing (WES) began to be adopted in children with severe short stature, and occasionally led to identification of NSSD in children with mild phenotypes.

For the purpose of this study, all identified variants were re-classified according to the latest ACMG/AMP 2015 guideline [37], utilising the Franklin platform (Genoox, Tel Aviv, Israel; https://franklin.genoox.com), incorporating phenotypic data when available. Only P or LP variants in NSSD-associated genes were included in the final cohort; variants of uncertain significance (VUS) were excluded.

Genetic testing was performed in several certified laboratories across the country; in this retrospective study, the specific methods used in each centre were not uniformly documented, and our role was limited to retrospective reclassification of the reported variants.

### Statistical analysis

Descriptive and inferential statistics were used to evaluate growth trajectories and genotype-specific differences. The significance level was set at *p* < 0.05. Paired parental height SDS values were compared using the Wilcoxon signed-rank test. The relationship between final height SDS and midparental, paternal, and maternal height SDS was assessed using Pearson’s correlation coefficient. In addition to nonparametric tests, regression analyses of genotype and phenotype correlations were performed, with growth at individual time points as the outcome, controlling for sex, age, and height (SDS) at the start of GH treatment, the stimulated GH, cardiological findings, and MPH (SDS). The comparison of the model including gene information to a model without this predictor using an *F*-test and on 95% confidence intervals for individual predictors was the main goal of the regression analyses. Patients with missing predictor/outcome values were excluded on a per-model basis. One potential problem when using the complete data set is the treatment of rare genotypes. Of note, pairwise nonparametric comparisons between common genotypes were not affected by the inclusion of rare genotypes in the data (these were ignored in the comparisons). For linear regression analyses, sensitivity analyses using only the two most common genotypes (*PTPN11*, *SOS1*) were performed. Statistical analyses and graphical outputs were performed using GraphPad Prism, version 5.02 (GraphPad Software, San Diego, CA, USA) and R version 4.5.1.

Missing data were present for some variables (e.g. parental heights available for 99/101 families, GH stimulation testing for 48/101 patients, menarche age for 13 girls, and final height for 23/101 patients). No imputation was performed. Patients with missing predictor or outcome values were excluded on a per-analysis basis.

The study was conducted in accordance with the Declaration of Helsinki and was approved by the joint Ethics Committee of the Second Faculty of Medicine, Charles University, and University Hospital Motol on June 12, 2024, under approval number EK–279.38/24. Written informed consent for data collection and registry participation was obtained as part of REPAR enrolment.

## Results

### Parental heights, perinatal data, and GH secretory status

**Fig. 2:**
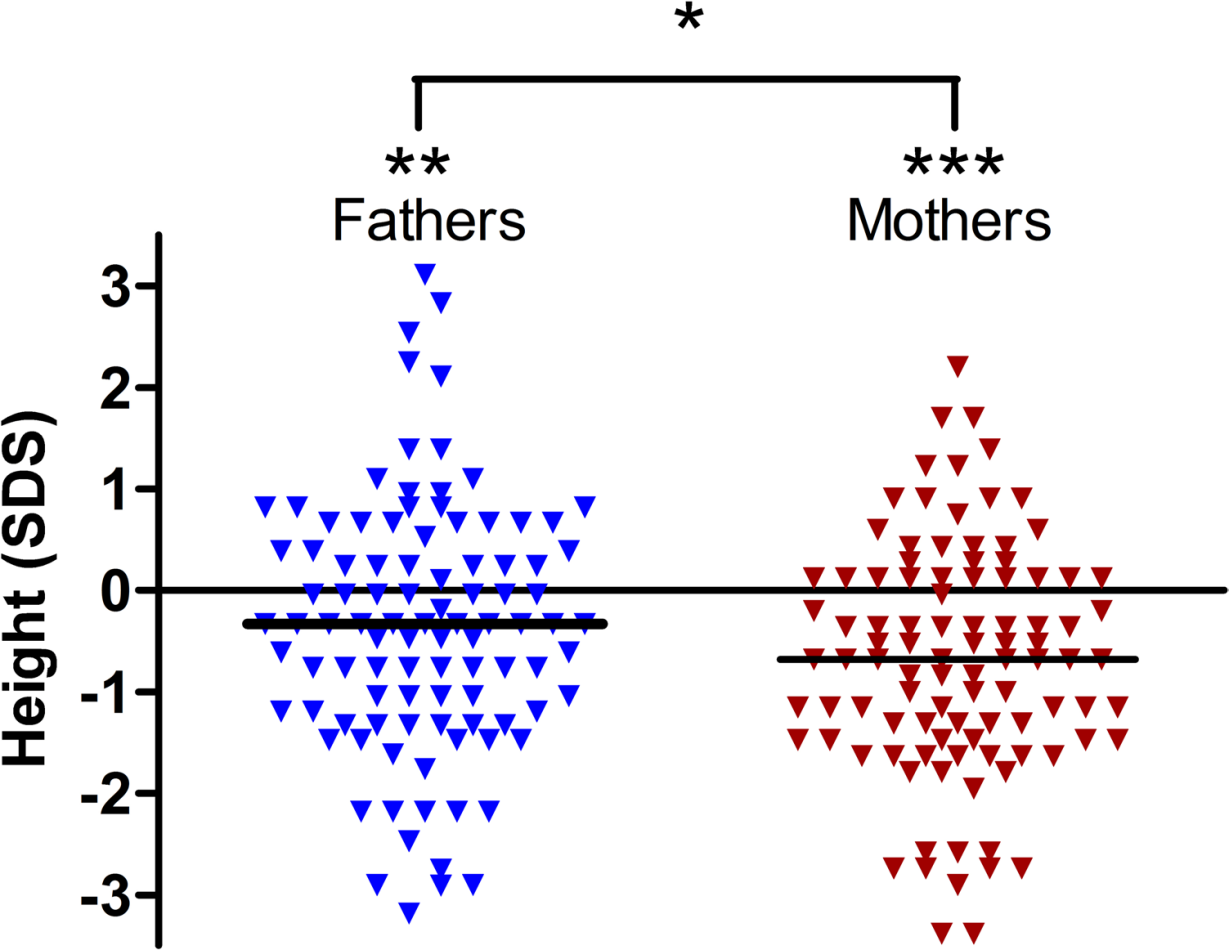
Height SDS of fathers and mothers of included patients. *n* = 99 parental pairs; the horizontal line indicates the median value. Both mothers and fathers are shorter if compared to population standards. The height-SDS in mothers is lower than in fathers. * *p* < 0.05; ** *p* < 0.01; *** *p* < 0.001

Parental height was below average, with a median of 178.0 cm (− 0.33 SDS [IQR − 1.19; 0.39]) in fathers (*p* < 0.01) and 163.0 cm (− 0.68 SDS [IQR − 1.47; 0.12]) in mothers (*p* < 0.001). Height-SDS in mothers was lower than in fathers (*p* < 0.05) (Fig. 2).

**Fig. 3:**
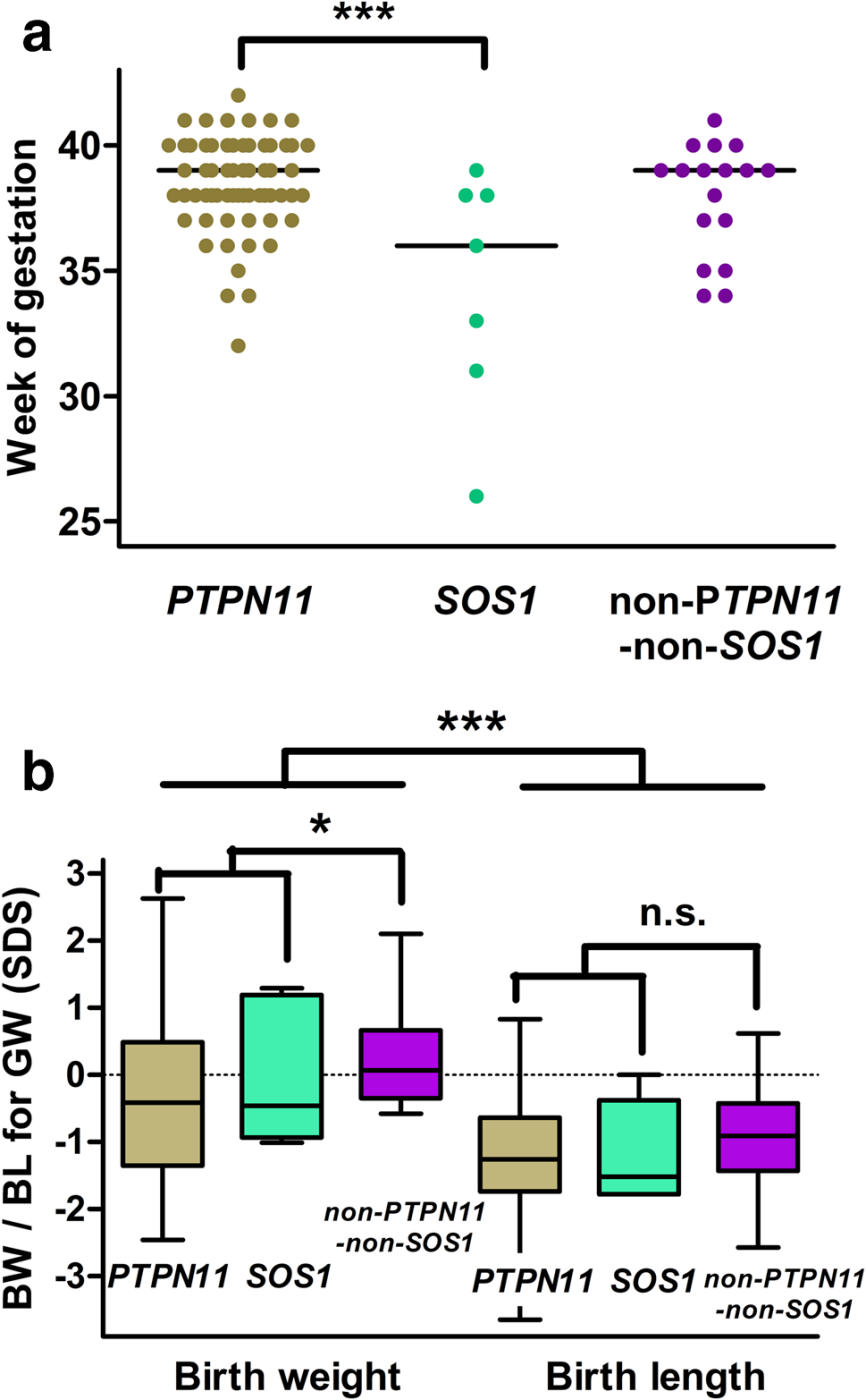
Perinatal data of genetically confirmed NSSD cohort. **A** Gestational week at birth by genotype; the horizontal line indicates the median value. **B** Size at birth (birth weight and birth length corrected for gestational age) in patients with *PTPN11*-NS, *SOS1*-NS, and other genotypes (median; IQR; range). *p < 0.05; ***p < 0.001; n.s.—not significant

In general, the children with NSSD were born at term (median gestational age: 39 weeks [37.0; 40.0]). Interestingly, the subgroup with *SOS1*-NS were born significantly earlier with median gestational age: 36 weeks [31; 37] compared to 39 weeks [38; 40] in the predominant *PTPN11-*NS sub cohort (Fig. 3A).

Children in the cohort were smaller at birth with a median birth length SDS of − 1.23 [IQR − 1.74; − 0.57] and birth weight SDS of − 0.29 [IQR − 1.1; 0.54] (*p* < 0.0001) compared to the general population of neonates, indicating the restriction of intrauterine longitudinal skeletal growth. Genotype-specific analysis revealed significantly lower birth weight SDS in children with *PTPN11*-NSSD and *SOS1*-NSSD variants compared to those with other genotypes (*p* < 0.05); a similar trend (not significant) was revealed in birth length SDS (Fig. 3B).

GH stimulation testing was performed in 48/101 patients, mostly in some of those diagnosed and treated prior to the approval of GH therapy for NS in Europe (before 2020). The peak GH reached 7.9 µg/L [IQR 5.4; 11.2], with 18 children having the stimulated peak GH below 7.0 µg/L, 15 children between 7.0 and 10.0 µg/L (‘partial’ growth hormone deficiency), and 15 children over 10.0 µg/L. GH secretory status, as assessed by stimulation testing, did not correlate with either the growth retardation before treatment or with the response to GH therapy. It seems to be a redundant investigation in NSSD after GH became an approved therapy for these children.

### First 5 years of GH therapy

**Fig. 4:**
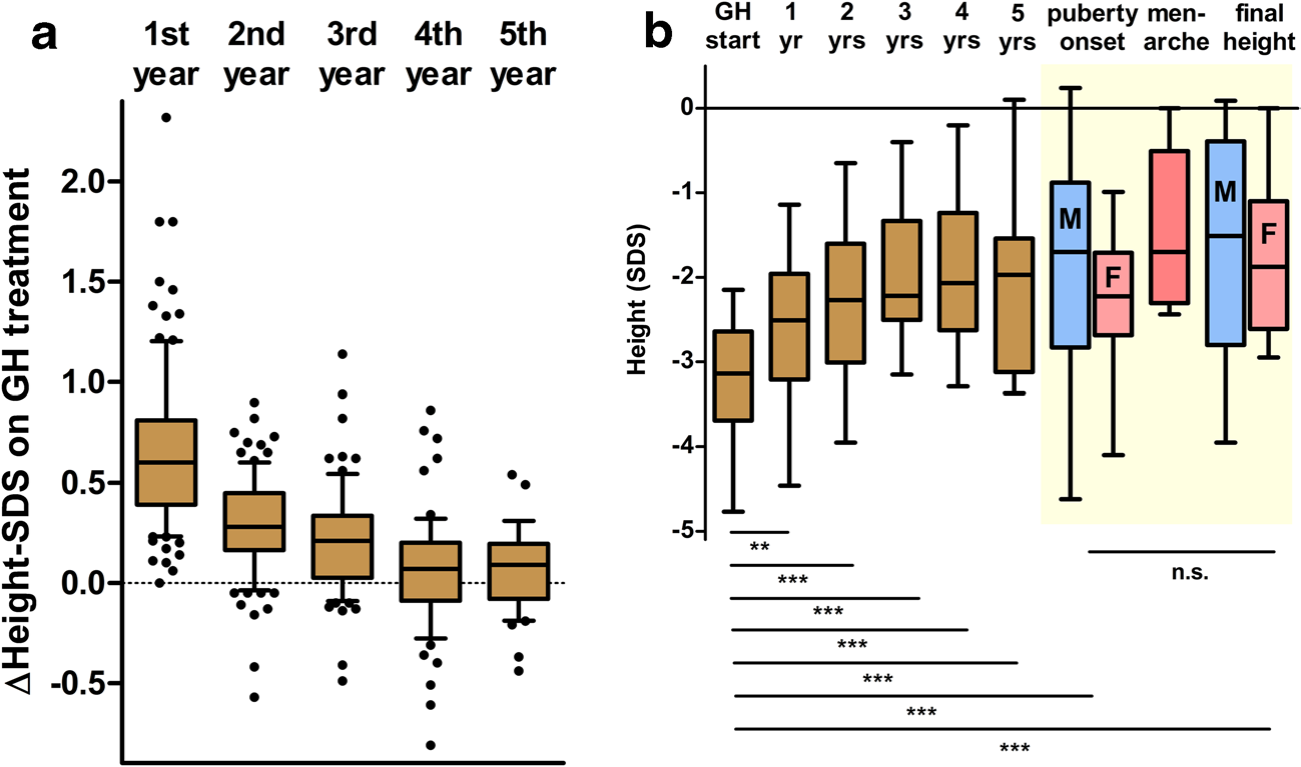
Yearly increments of height within the first 5 years of GH therapy. **A** Annual height increment (Δheight-SDS) in the whole cohort within the first five years of GH administration (mean; SEM). **B** Longitudinal height-SDS data in patients who already achieved final height (*n* = 23; Yellowish area highlights the pubertal period where data on males (*n* = 11) and females (*n* = 12) were plotted separately. **p < 0.01; ***p < 0.001; n.s.—not significant)

Annualised changes in height SDS (Δheight-SDS) in patients who completed each treatment year are shown in Fig. 4A. The median annual gain in height SDS was 0.61 [IQR 0.39; 0.81] in the first year (completed in all 101 patients), 0.28 [IQR 0.16; 0.45] in the second year (completed in 92), 0.21 [IQR 0.03; 0.34] in the third year (completed in 77), 0.07 [IQR − 0.09; 0.2] in the fourth year (completed in 63), and 0.09 [IQR − 0.08; 0.2] in the fifth year (completed in 41).

The median height SDS increased from − 2.92 [IQR − 3.64; − 2.47] at treatment initiation to − 1.97 [IQR − 2.80; − 1.42] after 5 years, with the greatest gain occurring during the first year. The yearly data are summarised in Table 1.

**Table 1:** Longitudinal height data

	GH start		Year 1	Year 2	Year 3	Year 4	Year 5	At onset of puberty (males; *n* = 11)		At onset of puberty (females; *n* = 12)		At menarche (*n* = 11)		At final height (*n* = 23)	
	Age (years [median, IQR])	Height-SDS (median [IQR]; mean (SEM))	(Height-SDS (median [IQR]; mean (SEM))	(Height-SDS (median [IQR]; mean (SEM))	(Height-SDS (median [IQR]; mean (SEM))	(Height-SDS (median [IQR]; mean (SEM))	(Height-SDS (median [IQR]; mean (SEM))	Age (years [median, IQR])	Height-SDS (median [IQR]; mean (SEM))	Age (years [median, [IQR])	Height-SDS ((median [IQR]; mean (SEM))	Age (years [median, IQR])	Height-SDS (median [IQR]; mean (SEM))	Age (years [median, IQR])	Height-SDS (median [IQR]; mean (SEM))
Whole cohort	6.4 [3.8; 9.5]	− 2.92 [− 3.64; − 2.47] − 3.04 (0.09)	− 2.35 [− 3.03; − 1.78] − 2.39 (0.09)	− 2.07 [− 2.86; − 1.52] − 2.15 (0.11)	− 1.83 [− 2.65; − 1.33] − 2.00 (0.12)	− 1.9 [− 2.67; − 1.24] − 1.99 (0.13)	− 1.97 [− 2.81; − 1.42] − 2.12 (0.17)	NA	NA	NA	NA	NA	NA	NA	NA
Final height subcohort (*n* = 23)	8.8 (5.8; 11.7)	− 3.14 [− 3.69; − 2.64] − 3.21 (0.14)	− 2.51 [− 3.21; − 1.96] − 2.61 (0.16)	− 2.27 [− 3.01; − 1.60] − 2.29 (0.18)	− 2.22 [− 2.50; − 1.34] − 1.95 (0.18)	− 2.07 [− 2.63; − 1.24] − 1.95 (0.19)	− 1.97 [− 3.12; − 1.54] 1.94 (0.26)	12.0 [11.3; 12.9]	− 1.70 [− 2.83; − 0.88] − 1.96 (0.41)	13.2 [11.8; 14.4]	− 2.23 [− 2.67; − 1.71] − 2.32 (0.25)	15.7 [13.8; 17.2]	− 1.70 [− 2.30; − 0.64] − 1.71 (0.28)	17.3 [16.0; 18.9]	− 1.68 [− 2.65; − 0.41] − 1.68 (0.24)

*NA* not applicable

### Pubertal milestones and final height

Twenty-three patients (12 girls and 11 boys) already achieved final height. The median final height-SDS was − 1.68 [IQR − 2.65; − 0.41]. Their average height loss related to midparental height was 9.7 cm. Notably, final height correlated moderately with maternal height-SDS (*r* = 0.62, *p* < 0.01), while no correlation was found with paternal height-SDS (*r* = 0.07, *p* = 0.76). Further details, including longitudinal height-SDS progression and height at key pubertal milestones, are displayed in Fig. 4B and in Table 1.

### Genotype-specific differences in GH response

GH treatment response was analysed across genetic subgroups. No statistically significant differences in cumulative height SDS gain at any timepoint were found between children with *PTPN11*-NS, *SOS1*-NS, and other genotypes using both nonparametric tests and regression analyses controlling for sex, age, and height (SDS) at the start of GH treatment, the stimulated GH, cardiological findings, and MPH (SDS). No statistically significant differences in GH-associated height SDS gain were observed across the genetically defined forms of NSSD; however, given the sample size, the analyses performed could not rule out a potential difference of approximately 0.5 SDS in at least one direction between *PTPN11* and most of the other genes studied, with the exception of the *SOS1*, *RAF1*, and *SHOC2* genes, for which such differences in SDS could be excluded for growth in the first and second year of therapy. For *SOS1*-NS, the 95% confidence interval for the difference from *PTPN11*-NS was − 0.20 to 0.34 SDS for the first year and − 0.19 to 0.22 SDS for the second year, i.e. no differences greater than approximately one quarter of the SDS were found. For a comprehensive overview of numeric clinical data stratified by causative genes, see Supplementary Table 2.

### Cardiac phenotype and growth outcomes

Cardiac phenotype was available in all patients and stratified into five groups: pulmonary valve stenosis (*n* = 31), hypertrophic cardiomyopathy (*n* = 3), combined pulmonary valve stenosis and hypertrophic cardiomyopathy (*n* = 8), other congenital heart defect (*n* = 18), and normal cardiac findings (*n* = 41). The association between genotype and cardiac phenotype was also summarised in Supplementary Table 1. When correlating cardiac subgroups with growth trajectories and GH treatment response, no statistically significant differences were observed.

## Discussion

This study provides a comprehensive evaluation of GH treatment outcomes in a genetically confirmed, registry-based nationwide cohort of children with NSSD. While most earlier studies relied on clinically diagnosed or only partially genotyped populations [27–31, 38–40], several more recent publications have analysed growth trajectories in fully genotyped NS cohorts [14–16]. However, these studies primarily focused on natural growth patterns or included only limited GH treatment data. To our best knowledge, this is the first study to combine a genetically defined cohort with longitudinal GH therapy outcomes across different genotypes, allowing for robust analysis of genotype-specific responses and early growth characteristics.

While some cohorts reported birth parameters within the normal range [22], others documented a modest but significant reduction in birth length [14–16, 20, 21]. Our findings therefore support the view of a mild but consistent intrauterine growth restriction. In the Czech Republic, birth length is routinely recorded as part of standard neonatal assessment, which enabled a more accurate evaluation of prenatal longitudinal skeletal growth.

It is not surprising that the parental heights of children with NSSD were shorter than the population mean. All forms of NSSD follow autosomal dominant inheritance, though with a substantial addition of de novo pathogenic variants [41]. Croonen et al. found that maternal height was a significant determinant of early postnatal growth in NS [22], consistent with our observation that maternal height correlated more strongly than paternal height with final height. In our cohort, the observed discrepancy between maternal and paternal height correlations with final height could be consistent with reports of more frequent maternal transmission. Men with NS are also more often affected by subfertility than women, which may correspond with our observation [42–44]. Nevertheless, any such interpretation remains speculative in the absence of parental genetic testing [34, 41]. Delayed pubertal onset is generally considered a feature of the NS phenotype; our data suggest that this delay is apparent more in girls than in boys. The mechanism behind this sex difference remains unclear, and due to the lack of detailed hormonal or staging data, further investigation is warranted.

A reduced growth hormone response in patients with *PTPN11* mutations was initially suggested by Binder et al. [25], prompting further investigation into genotype-specific treatment effects. However, subsequent studies including Choi et al. [26] and the recent multicentre study by Şıklar et al. [13], both based on genetically tested cohorts, did not confirm a significant association between genotype and GH response. Similarly, our data did not reveal any meaningful genotype-related differences in treatment outcomes. However, this does not mean that we can completely rule it out due to the size and distribution of gene findings in our dataset. Minor differences in SDS height after the first year of treatment, which did not reach statistical significance, were observed in some genetic subgroups. Taken together, current findings suggest that, while genetic background may influence baseline growth characteristics at birth and maybe thereafter, its impact on GH therapy response remains unclear. Larger national or combined datasets of genetically well-characterised individuals with NSSD would help clarify the effect of the disrupted gene on response to GH treatment.

A noteworthy strength of this study lies in its integration within the national REPAR registry, which prospectively collects longitudinal data on all paediatric GH recipients in the Czech Republic. The use of standardised national growth references [36] and strict molecular inclusion criteria enhance the generalizability of findings within similar populations. Notably, our cohort represents one of the few population-based, multicentre collections of genetically confirmed NSSD patients to date, providing a strong basis for analysing genotype-specific growth trajectories while substantially reducing referral and ascertainment biases.

Given that GH therapy is fully reimbursed through the Czech public health insurance system, financial considerations do not limit access to treatment. This minimises potential socioeconomic bias in patient selection and supports the representativeness of our country-based cohort.

Several limitations must be acknowledged that are linked to the retrospective observational character of the study with the acquisition of patients over a longer period of time. Prior to the formal European approval of GH treatment for Noonan syndrome in 2020, patients were commonly treated under alternative indications such as growth hormone deficiency or small for gestational age. This may have introduced minor selection bias. In addition, data on parental genotyping were not available over the whole study cohort. We may only conclude that in some patients, one of the parents carried a pathogenic gene variant and would presumably be shorter. This makes a detailed analysis of mid-parental height on treatment outcomes difficult.

The predominance of *PTPN11* variants (75%) in our cohort is slightly higher than in other fully genotyped NS populations, where frequencies between 53 and 71% have been reported [14–16]. Although this may partially reflect the natural variation in genotype distributions across studies, the relatively high *PTPN11* representation could also result from earlier referral or diagnostic patterns. Importantly, the inclusion of only genetically confirmed cases ensured internal consistency and reliable genotype classification throughout the study.

Evidence from previous studies further suggests that some individuals with milder phenotypes or non-classical presentations of RASopathies were only identified through untargeted genetic screening [45, 46], highlighting how both clinical recognition and the real-life availability of genetic testing influence the representation of specific genotypes in NSSD cohorts.

To conclude, this study likely represents the first genotype-stratified evaluation of growth hormone therapy outcomes in a large country-based cohort of genetically confirmed Noonan syndrome patients. Despite initial differences in birth parameters and baseline growth, no statistically significant genotype-related differences in height gain during GH therapy were observed in this cohort. Our data confirm a mild but consistent intrauterine growth restriction, particularly in children with *PTPN11* or *SOS1* variants. These findings contribute to a more accurate description of growth patterns in Noonan syndrome and other Noonan syndrome spectrum disorders and may help set realistic expectations for families and clinicians managing growth in affected children.

## Supplementary Information

Below is the link to the electronic supplementary material.

ESM 1 (DOCX 23.5 KB)

ESM 2 (DOCX 20.3 KB)

## Data Availability

The data that support the findings of this study are not openly available due to reasons of sensitivity and are available from the corresponding author upon reasonable request.

## Abbreviations

ACMG/AMP: American College of Medical Genetics and Genomics/Association for Molecular Pathology
CFC: Cardiofaciocutaneous
GH: Growth hormone
IQR: Interquartile range
LP: Likely pathogenic
NGS: Next-generation sequencing
NS: Noonan syndrome
NSSD: Noonan syndrome spectrum disorders
*PTPN11*-NS: Noonan syndrome caused by a pathogenic variant in *PTPN11* gene
REPAR: Registry of paediatric growth hormone recipients in Czechia
SDS: Standard deviation score
SEM: Standard error of the mean
*SOS1*-NS: Noonan syndrome caused by a pathogenic variant in *SOS1* gene
VUS: Variant of uncertain significance
WES: Whole exome sequencing
