# Supplementary material for: Noonan syndrome spectrum disorders in real life: patient characteristics and response to growth hormone therapy in a genetically defined single-country multicenter cohort
Source: Eur J Pediatr. 2026 Jan 24;185(2):102. doi: 10.1007/s00431-026-06764-2 (PMC12830400; doi:10.1007/s00431-026-06764-2)
Supplement: Supplementary file 1 — (DOCX 23.5 KB) [file 431_2026_6764_MOESM1_ESM.docx]

**Supplementary Table 1. List of genetic and cardiological findings**

| **Gene** | **Nucleotide Variant (HGVS, c.)** | **Protein Variant** | **Number of patients** | **Cardiac phenotype** |
| --- | --- | --- | --- | --- |
| *PTPN11* | c.5C>T | p.(Thr2Ile) | 4 | N (4) |
| *PTPN11* | c.124A>G | p.(Thr42Ala) | 1 | N |
| *PTPN11* | c.172A>G | p.(Asn58Asp) | 4 | PS (1); CoA (1); N (2) |
| *PTPN11* | c.178G>A | p.(Gly60Ser) | 1 | PS |
| *PTPN11* | c.181G>A | p.(Asp61Asn) | 1 | PS |
| *PTPN11* | c.182A>G | p.(Asp61Gly) | 1 | PS+ASD |
| *PTPN11* | c.185A>C | p.(Tyr62Ser) | 1 | PS |
| *PTPN11* | c.188A>G | p.(Tyr63Cys) | 5 | PS+VSD (1); PS (2); N (2) |
| *PTPN11* | c.211G>T | p.(Ala72Ser) | 1 | PS+CoA+VSD |
| *PTPN11* | c.211T>A | p.(Phe71Ile) | 1 | N |
| *PTPN11* | c.214G>T | p.(Ala72Ser) | 1 | PS+ASD |
| *PTPN11* | c.215C>G | p.(Ala72Gly) | 3 | PS (1); ASD+PI+TI (1); ASD (1)jirova |
| *PTPN11* | c.228G>T | p.(Glu76Asp) | 1 | N |
| *PTPN11* | c.236A>G | p.(Gln79Arg) | 3 | PS (1); PS+ASD (1); N (1) |
| *PTPN11* | c.317A>C | p.(Asp106Ala) | 1 | PS |
| *PTPN11* | c.417G>C | p.(Glu139Asp) | 6 | PS (2); N (4) |
| *PTPN11* | c.781C>T | p.(Leu261Phe) | 1 | PS |
| *PTPN11* | c.785T>G | p.(Leu262Arg) | 3 | N (3) |
| *PTPN11* | c.794G>A | p.(Arg265Gln) | 3 | ASD (1); N (2) |
| *PTPN11* | c.802G>A | p.(Gly268Ser) | 1 | N |
| *PTPN11* | c.802G>T | p.(Gly268Cys) | 1 | N |
| *PTPN11* | c.836A>G | p.(Tyr279Cys) | 1 | N |
| *PTPN11* | c.844A>G | p.(Ile282Val) | 1 | MI |
| *PTPN11* | c.853T>C | p.(Phe285Leu) | 1 | PS, ASD, HCMP |
| *PTPN11* | c.922A>G | p. (Asn308Asp) | 12 | PS+TI (1); PS (3); N (8) |
| *PTPN11* | c.923A>G | p. (Asn308Ser) | 2 | PS+HCMP+ASD (1); PS (1) |
| *PTPN11* | c.1403C>T | p.(Thr468Met) | 3 | PS (1); ASD (1); N (1) |
| *PTPN11* | c.1472C>T | p.(Pro491Leu) | 2 | AI (2) |
| *PTPN11* | c.1492C>T | p.(Arg498Trp) | 1 | N |
| *PTPN11* | c.1504T>A | p.(Ser502Thr) | 1 | PS |
| *PTPN11* | c.1507 G> | p.(Gly503Arg) | 1 | N |
| *PTPN11* | c.1508G>A | p.(Gly503Glu) | 1 | N |
| *PTPN11* | c.1510A>G | p.(Met504Val) | 5 | PS (2); PS+HCMP+ASD+VSD (1); FoA (1); N (1) |
| *PTPN11* | c.1528C>G | p.(Gln510Glu) | 1 | PS+HCMP |
| *SOS1* | c.797C>A | p.(Thr266Lys) | 2 | PS+AI (1); PS (1) |
| *SOS1* | c.1288G>C | p.(Asp430His) | 1 | PS |
| *SOS1* | c.1294T>C | p.(Trp432Arg) | 1 | PS+MI |
| *SOS1* | c.1310T>C | p.(Ile437Thr) | 1 | N |
| *SOS1* | c.1655G>A | p.(Arg552Lys) | 2 | PS+HCMP (1); MI+AI (1) |
| *RAF1* | c.770C>T | p.(Ser257Leu) | 2 | PS+HCMP+ASD+MI (1); HCMP+MI (1) |
| *RAF1* | c.788T>A | p.(Val263Asp) | 1 | HCMP |
| *RAF1* | c.1423T>C | p.(Phe475Leu) | 1 | N |
| *KRAS* | c.17T>C | p.(Leu6Pro) | 1 | ASD |
| *KRAS* | c.179G>T | p.(Gly60Val) | 1 | FoA+TI |
| *KRAS* | c.458A>T | p.(Asp153Val) | 1 | PS+HCMP |
| *BRAF* | c.770A>G | p.(Gln257Arg) | 2 | PS+AS+ASD+MI+HCMP (1); ASD (1) |
| *HRAS* | c.186_206dup | p.(Glu62_Arg68dup) | 1 | PS |
| *HRAS* | exon 1–6 deletion |  | 1 | N |
| *SOS2* | c.1867C>T | p.(Arg623Cys) | 1 | N |
| *SOS2* | c. 1127C>G | p(Thr376Ser) | 1 | N |
| *SHOC2* | c.4A>G | p.(Ser2Gly) | 2 | HCMP+VSD+MI+CoA (1); N (1) |
| *LZTR1* | c.742G>A | p.(Gly248Arg) | 1 | PS |
| *MAP2K1* | c.199G>A | p.(Asp67Asn) | 1 | N |
| *NRAS* | c.34G>C | p.(Gly12Arg) | 1 | MI |

AS - aortic stenosis; ASD - atrium septum defect; CoA - aortic coarctation; FoA - foramen ovale apertum; HCMP - hypertrophic cardiomyopathy; MI - mitral insufficiency; N - normal findings; PI - pulmonary insufficiency; PS - pulmonary stenosis; TI - tricuspidal insufficiency; VSD - ventricle septum defect
