# Supplementary material for: Noonan syndrome spectrum disorders in real life: patient characteristics and response to growth hormone therapy in a genetically defined single-country multicenter cohort
Source: Eur J Pediatr. 2026 Jan 24;185(2):102. doi: 10.1007/s00431-026-06764-2 (PMC12830400; doi:10.1007/s00431-026-06764-2)
Supplement: Supplementary file 2 — (DOCX 20.3 KB) [file 431_2026_6764_MOESM2_ESM.docx]

**Supplementary Table 2. Overview of numeric clinical data according to causative genes**

Patient characteristics and results of GH therapy displayed as numeric values according to the causative gene. All data are displayed as medians and interquartal ranges [IQR]. N/A - not applicable; SDS - standard deviation score.

| Gene | Number of  patients | Midparental height  (cm) | Birth lenght  (SDS) | Birth  weight (SDS) | Height at GH start (SDS) | Height at Year 1 of therapy  (SDS) | Height at Year 5 of therapy  (SDS) | Final Height (SDS) |
| --- | --- | --- | --- | --- | --- | --- | --- | --- |
| *PTPN11* | 76 | 169 [165;176] | -1.26 [-1.74;-0.64] | -0.41 [-1.35;0.49] | -2.88  [-3.66; -2.42] | -2.34 [-2.91; -1.76] | -1.99 [-2.69; -1.25] | -1.88 [-2.66; -1.14] |
| *SOS1* | 7 | 175 [165;177] | -1.52 [-1.73;-0.74] | -0.46 [-0.76;0.48] | -2.92 [-3.89;-2.52] | -2.12 [-3.24;-1.78] | -2.24 [-2.95;-1.45] | -1.36 [-1.94;-0.77] |
| *RAF1* | 4 | 175 [170;177] | -0.94 [-1.14;-0.79] | 0.05 [-0.05;0.19] | -2.99 [-3.46;-2.72] | -2.40 [-2.99;-2.02] | -2.86 [-3.30;-2.41] | N/A |
| *KRAS* | 3 | 176 [172;179] | -0.55 [-0.73;0.04] | 1.05 [0.88;1.58] | -2.70 [-2.93;-2.42] | -2.47 [-2.60;-2.08] | -1.83 [-1.84;-1.82] | N/A |
| *BRAF* | 2 | 172 [169;175] | -1.09 [-1.48;-0.70] | -0.00 [-0.15;0.14] | -4.16 [-5.03;-3.30] | -3.25 [-4.11;-2.39] | -6.17 | N/A |
| *HRAS* | 2 | 170 [166;174] | -2.36 [-2.47;-2.25] | -0.42 [-0.43;-0.41] | -3.79 [-4.09;-3.48] | -3.21 [-3.35;-3.06] | -1.7 | -2.8 |
| *SOS2* | 2 | 175 [172;179] | -1.31 [-1.78;-0.83] | -0.48 [-0.94;-0.01] | -2.95 [-3.59;-2.31] | -1.83 [-2.51;-1.14] | -0.84 [-1.77;0.10] | -0.16 [-0.41;0.09] |
| *SHOC2* | 2 | 171 [170.5;171] | -0.37 [-0.95;0.21] | 0.08 [-0.58;0.74] | -3.23 [-3.55;-2.90] | -2.47 [-3.08;-1.85] | -2.33 | N/A |
| *LZTR1* | 1 | 178 | 0.04 | 0.27 | -3.23 | -2.9 | N/A | N/A |
| *MAP2K1* | 1 | 166 | -1.23 | -0.48 | -3.43 | -2.09 | N/A | N/A |
| *NRAS* | 1 | 164 | -1.41 | 0.62 | -4.06 | -3.49 | N/A | N/A |
